# Variation in behavioural maturation in tropical honey bees corresponds with hormonal and molecular differences

**DOI:** 10.1242/jeb.251399

**Published:** 2026-04-23

**Authors:** Sruthi Unnikrishnan, Deepika Bais, Ashwin Suryanarayanan, Aridni Shah, Axel Brockmann

**Affiliations:** National Centre for Biological Sciences, Tata Institute of Fundamental Research (TIFR), Bengaluru, Karnataka 560065, India

**Keywords:** Asian honey bees, Juvenile hormone, Comparative study, Foraging, *Apis florea*, *Apis cerana*

## Abstract

Division of labour in honey bees is based on a process of behavioural development in which the worker bee successively performs different tasks at different ages. Workers start with tasks within the nest and move on to become foragers. In *Apis mellifera*, juvenile hormone and vitellogenin are major drivers of this behavioural maturation, which is accompanied by changes in brain physiology, including changes in neuronal gene expression and synaptic connections. Based on this detailed knowledge, we asked whether and how major characteristics of the behavioural maturation process vary among two tropical Asian honey bee species, the phylogenetically ancestral open-nesting *Apis florea* and the cavity-nesting *Apis cerana*, a sister species to *Apis mellifera*. Our behavioural studies showed that workers of *A. florea* exhibited a slower pace of behavioural maturation than *A. cerana*, with greater individual variation in the age at onset of foraging. In both species, the expression pattern of juvenile hormone and foraging-associated transcription factors broadly mirrored those reported for *A. mellifera*. In contrast, expression dynamics of vitellogenin and nurse-associated transcription factors in both species did not show the clear age- or task-related pattern as reported for *A. mellifera*. Notably, workers of *A. florea* consistently exhibited substantially higher vitellogenin expression levels than did *A. cerana* workers. Based on our findings, we propose that evolution of accelerated behavioural maturation in cavity-nesting species is primarily attributed to changes in the temporal dynamics of juvenile hormone signalling, whereas vitellogenin levels might vary according to different social functions.

## Teaser

**Summary:** Studies on onset of foraging in workers of the tropical honey bees *A. cerana* and *A. florea* indicate evolutionary changes in developmental pace and distinct hormonal and molecular adaptations.

## INTRODUCTION

Division of labour among individuals is a hallmark of eusocial insects (Beshers and Fewell, 2001; Wilson, 1985; Woodard et al., 2011). It is either based on rigid physical castes with individuals performing tasks linked to their specialised morphological phenotype or temporal castes with morphologically similar individuals performing tasks according to their age (Beshers and Fewell, 2001; Michener, 1974; Noirot and Bordereau, 1991; Oster and Wilson, 1978). This age-based division of labour or age polyethism is a form of behavioural maturation, similar to juvenile–adult development in vertebrates (Noirot and Bordereau, 1991), in which hormones regulate a succession of behavioural changes by means of changing the form and physiology of the body and brain circuits (Barredo et al., 2021; Robinson, 1992).

The Western honey bee, *Apis mellifera*, is one of the most well-studied eusocial species for understanding the social and physiological mechanisms underlying worker behavioural maturation (Huang and Robinson, 1992; Huang et al., 1994; Whitfield et al., 2006). Workers of *A. mellifera* live for ∼6 weeks during the summer season. The first part of their lives is spent inside the hive, performing tasks within the colony, and the second part is spent outside as foragers, collecting food for the colony (Free, 1965; Lindauer, 1952; Prado et al., 2020; Seeley, 1982). This onset of foraging is one of the most striking changes in the workers' lives and has often been used as an experimental paradigm to study the underlying hormonal and molecular processes involved in age polyethism (Huang and Robinson, 1992, 1996; Kolmes, 1985; Whitfield et al., 2003).

The key pacemaker of the onset of foraging is juvenile hormone (JH). JH titres in the haemolymph are significantly different between nurse bees and foragers and increase with age (Huang and Robinson, 1992; Huang et al., 1994; Jassim et al., 2000; Rutz et al., 1976). Treatment with methoprene, a JH analogue, accelerates the onset of foraging (Jaycox, 1976; Jaycox et al., 1974; Robinson, 1985). However, allatectomised bees (bees in which the corpora allata, which is the production centre of JH, was removed) still developed into foragers, albeit with a delay, indicating that JH is not necessary for initiating the behavioural maturation of honey bee foragers (Sullivan et al., 2003). In fact, there is accumulating evidence that, in addition to JH, other hormones, peptides involved in feeding behaviour, such as insulin-like peptide (Ilp-1) and neuropeptide Y-like (NPF), and neuromodulators, such as octopamine, affect the onset of foraging (Ament et al., 2008, 2011; Reim and Scheiner, 2014; Schulz et al., 2002). Furthermore, the egg-yolk precursor protein, vitellogenin (Vg), appears to affect and regulate behavioural maturation and longevity in the sterile worker caste (Nelson et al., 2007). Vg titres are high in nurse bees and decline with age in summer bees, whereas they are constantly heightened in winter bees that do not forage and exhibit an extended life span (Engels, 1974; Fluri et al., 1977). Nurse bees use Vg to synthesise brood food, and winter bees use it as a metabolic energy reserve (Amdam et al., 2003, 2005), and it has beneficial effects on cellular immunity and antioxidant function (Amdam et al., 2004; Seehuus et al., 2006). Finally, Amdam and Omholt (2003) proposed that Vg might be directly involved in the temporal dynamic of behavioural maturation via mutual inhibitory interaction with JH. High levels of the JH analogue pyriproxyfen were shown to inhibit Vg synthesis (Pinto et al., 2000), and knocking down Vg led to precocious foraging and increase in JH titres (Guidugli et al., 2005; Marco Antonio et al., 2008; Nelson et al., 2007).

In the past two decades, since the sequencing of the honey bee genome, studies on age polyethism have focused on studying brain gene expression changes involved in the onset of foraging (Ben-Shahar et al., 2002; Grozinger et al., 2003; Whitfield et al., 2003). Studies estimate that ∼1000 genes are differentially expressed in the brains of nurses and foragers, and their expression is regulated by a set of at least 15 transcription factors (Chandrasekaran et al., 2011; Jones et al., 2020; Khamis et al., 2015; Whitfield et al., 2003). Some of these identified transcription factors are associated with nursing behaviours, such as broad complex (BR-C) and nautilus/MyoD1, and others with foraging behaviours, e.g. ultraspiracle (usp) and early growth-response 1 (egr-1) (Ament et al., 2012; Barchuk et al., 2004; Chandrasekaran et al., 2011; Hamilton et al., 2019; Khamis et al., 2015).

Our detailed knowledge of worker behavioural maturation in *Apis mellifera* and the recent sequencing of the genomes of the three major Asian honey bees, *Apis cerana*, *Apis dorsata* and *Apis florea* (Diao et al., 2018; Fouks et al., 2021; Karpe et al., 2016; Oppenheim et al.; Park et al., 2015; *A. florea*, NCBI RefSeq assembly GCF_000184785.3) open the possibility to exploring evolutionary variation and plasticity in the regulation of age-dependent division of labour within a small monophyletic group of eusocial bees (Alexander, 1991; Sen Sarma et al., 2007; Smith, 2020). Honey bee species differ in their distribution range, nesting behaviour, and body and colony size. The phylogenetically ancestral dwarf bee, such as *A. florea*, and giant honey bee species, such as *A. dorsata* (also known as open-nesting honey bees), build nests comprising a single comb attached to tree branches, cliff sides or overhangs of human constructions. The workers of the colony form a curtain around the comb, such that it is protected from adverse environmental conditions, parasites and predators (Bhagavan et al., 2016; Dyer and Seeley, 1991; Ruttner, 1988; Seeley et al., 1982). These open-nesting honey bees are restricted to the tropics (Hepburn and Radloff, 2011; Smith, 2020). The phylogenetically derived cavity-nesting species, such as *A. mellifera* and *A. cerana*, construct nests of multiple combs preferentially inside cavities in tree trunks, crevices of rocks or buildings. Because the cavity protects the nest, these species generally do not form a curtain surrounding the combs (Seeley, 1983; Seeley and Morse, 1976). Cavity nesting allowed populations of *A. mellifera* and *A. cerana* to extend their distribution into temperate climates. Studying the effects of nesting behaviour on colony organisation in the different species, Seeley and colleagues proposed that nesting behaviour also affects behavioural maturation of workers and age at the onset of foraging (Dyer and Seeley, 1991; Seeley et al., 1982); however, genuine comparative studies are still lacking (Bhagavan and Brockmann, 2019; Huang et al., 2001; Rahman et al., 2025).

In the current study, we provide a comparative study of behavioural maturation in tropical colonies of two species, an open nesting species, *A. florea*, and a cavity-nesting species *A. cerana*, in Bengaluru, South India. Our objectives were (1) to study whether there is a difference in behavioural maturation between open- and cavity-nesting honey bees, and (2) to understand whether tropical species follow the same patterns for behavioural maturation as the temperate species, *A. mellifera*. First, we studied the foraging behaviour to identify the age of onset of foraging in both species. Second, we investigated the temporal dynamics of JH and Vg in both species. We also studied the JH titres, and gene expression levels of *Vg* and *ilp-1* in nurses and foragers. Finally, we determined the gene expression levels of four transcription factors in nurses and foragers, two of which are associated with foraging behaviour, *usp* and *egr-1*, and two of which are associated with nursing behaviour, *BR-C* and *nautilus*. We obtained all colonies from Bengaluru Urban district to avoid any population differences that could arise from different locations (Bharath Kumar et al., 2024).

## MATERIALS AND METHODS

### Behavioural observations

#### Study area

The study was performed in Bengaluru Urban, Karnataka, India. In Bengaluru, the seasonal changes are not very pronounced with respect to its temperature, with the city experiencing relatively small temperature variations throughout the year. The average temperature in the coldest month of December is ∼21.4°C, while the warmest month, April, shows an average of 28**°**C. Even at the extremes, temperatures rarely fall below 15°C or rise above 34°C (https://th.climate-data.org/; https://weather-and-climate.com/). In general, flowering peaks in the pre-monsoon months (March–May), with a smaller secondary peak in winter (December–February) in Bengaluru Urban (Centre for Ecological Sciences, Indian Institute of Science, 2014). All the colonies of *A. cerana and A. florea* that were studied were obtained and observed in Bengaluru. We did not observe any major changes in honey bee nest initiation throughout the year for *A. cerana* and *A. florea*. We also mostly observed the two species in similar seasons to minimise environmental effects (Table S4).

##### Apis cerana

Four framed colonies of *Apis cerana* Fabricius 1793 were obtained from a local beekeeper and maintained on the campus of the National Centre for Biological Sciences, Tata Institute of Fundamental Research (TIFR), Bengaluru. Colonies were selected to be of similar size, apparent strength and brood composition, which are commonly good indicators of colony demographic state and are expected to reduce large differences in worker age structure. For the behavioural experiments, the colonies were split, and two frames with workers and the queen were transferred into an observation hive. We did not observe any strong effects on worker behavioural maturation when they were kept in observation hives, compared with regular hives, both in our study (see Fig. 2) and in previous work (Rahman et al., 2025). The brood frames of the colonies were kept inside incubators at a temperature of 33–34°C for 24 h. This allowed late-stage pupae to eclose as adults. These day-old bees were individually colour marked and released into their respective colonies. Only two colonies could be successfully studied; five colonies absconded before the end of the experiment and could not be observed completely. Out of the two colonies, for the first colony (Cerana 1), 77 marked day-old bees, and for the second colony (Cerana 2), 164 marked day-old bees, were released. Behavioural observations were performed to follow the behaviour of these marked bees across days until day 24. A video camera kept at the entrance of the observation hive for 2–4 h every day (times rotated every day between 08:00 h and 18:00 h to cover all active hours) until day 35 allowed us to study when the introduced bees started foraging. An individual with a foraging trip duration of above 2 min was considered a forager, which was also the minimum time taken by a pollen forager in these colonies. In addition, bees were also considered as foragers if they followed a dance or danced. In this manner, the age at first foraging trip or onset of foraging of each marked bee was identified. Cerana 1 was observed between December 2017 and February 2018, and Cerana 2 was observed between April and June 2019. A detailed table on nests collected for each study is provided in Table S4.

##### Apis florea

Colonies of *Apis florea* Fabricius 1787 were collected with the help of a local beekeeper and maintained on the campus of the National Centre for Biological Sciences, TIFR, Bengaluru. As *A. florea* naturally occurs as single-comb colonies, colonies with similarly sized combs of intermediate size were selected, avoiding very small or recently established colonies, to minimise extreme variation in colony demography. Comb areas with brood were collected from source colonies that were distinct from the experimental colonies and incubated at 33–34°C for 24 h to allow adult bees to eclose. For each experimental colony, all introduced/day-old workers originated from a single brood-source colony (i.e. brood from one source colony was introduced only into one experimental colony), and brood from different source colonies was not mixed within experimental colonies. These day-old bees were individually colour marked (100 bees for each colony) and introduced into the colonies. A video camera kept in front of the colonies recorded for 2–4 h (times rotated every day between 08:00 h and 18:00 h to cover all active hours) every day until the colony absconded. Using the same criteria as described above for identifying foragers, foraging trips and the onset of foraging of the marked bees was studied in *A. florea*. Out of 10 colonies, only two (Florea 1 and Florea 2) were successfully observed, as the remaining absconded within a short time. Florea 1 only absconded by day 70, and Florea 2 absconded by day 40. Florea 1 was observed between December 2018 and February 2019, and Florea 2 was observed from October to November 2018.

### Hormonal and genetic analysis

#### Sample collection

##### Age-related changes in JH titres and *Vg* expression

Day-old bees were individually marked and released (100 each) in the same manner as for behavioural observations, for two colonies of *A. cerana* (six frames each) and *A. florea*. The only difference was that *A. cerana* colonies were not transferred into observation hives as in the case of the behavioural analysis. Similarly to the behaviour observations, foraging behaviour was studied with the help of video cameras kept outside the colonies for 2–4 h every day until the end of the collection. Individuals were collected on days 0 (day-old bees), 5, 10, 18, 25, 30, 35, 40, 45 and 50 for measuring their JH titres in the haemolymph. We collected approximately four to five bees per colony for each age class collection. After haemolymph extraction, these bees were stored at −80°C until abdominal *Vg* gene expression levels were measured. The collections and observations of these colonies were performed simultaneously from November 2019 to January 2020. All four colonies used for JH analysis were different from the ones used for behavioural observations in the previous section. A list of colonies used for the different studies is provided in Table S4. JH was quantified using mass spectrometry (MS) based on its molecular mass and fragmentation pattern.

##### Nurse–forager comparison

Individual nurse bees and foragers were collected from six colonies each of *A. cerana* (five frames each) and *A. florea* in January 2020 based on the tasks they were observed to be performing irrespective of their age. Nurses were identified as those bees that inserted their heads into cells containing larvae (Bhagavan and Brockmann, 2019; Brouwers et al., 1987; Hepburn et al., 2014; Huang and Otis, 1991; Seeley, 1995; Seeley and Visscher, 1985). Foragers were identified based on the fact that they had pollen load when returning to the colony. A total of five nurse bees and five foragers per colony were collected, bringing a total of 60 bees for the study. Three colonies were used for measuring JH titres (total number of bees, 30) and the remaining three for measuring *Vg*, *ilp-1* and transcription factor gene expression levels (total number of bees, 30) (Table S4). Individuals collected (except those used for JH studies) were immediately stored at −80°C (all raw data are available at Unnikrishnan et al., 2024).

#### JH measurements

##### Extraction of haemolymph and sample preparation

The collected individuals were immediately anaesthetised on ice. Their antennae were cut using dissection scissors and the individuals were inverted and centrifuged, such that their haemolymph would flow out into collection tubes through the cut antennae (Ares et al., 2012). Then, 10 μl of this collected haemolymph from each individual bee was mixed with 22 μl methanol and 8 μl internal standard (juvenile hormone ethyl esterase, JHEE). JHEE was made by a transesterification process of JH following methods established by Scholl et al. (2014) and used as the internal standard. JH measured in this study was JH III. Hormone identity was confirmed using MS, with detection of a parent ion at ≈267 m/z in positive-ion mode and characteristic product ions at 147 and 109 m/z, consistent with JH III (hereafter referred to as JH). If we were unable to obtain 10 μl haemolymph from an individual bee, methanol was added to the amount that could be collected to make it up to 10 μl, so that the total volume of the mixture came to 40 μl. The samples were then sonicated for 2 min and centrifuged for 10 min at 14,000 rpm (∼16,000 ***g***). The supernatant was analysed using liquid chromatography–tandem mass spectrometry (LC/MS-MS).

##### LC/MS-MS conditions

A TSQ Vantage triple-stage quadrupole mass spectrometer (Thermo Fisher Scientific, San Jose, CA, USA), connected to an Agilent 1290 infinity series UHPLC system (Agilent Technologies India Pvt. Ltd, India) was used for LC/MS-MS. The column oven was set at 40°C and the autosampler tray at 4°C. Mobile phase solvent A was 10 mmol l^−1^ ammonium acetate with 0.1% formic acid, and mobile phase solvent B was methanol (100%). The chromatography was carried out in an 80 Å column [30×4.6 mm inner diameter (i.d.)], which was protected by a C18 guard column (4×2 mm i.d.), both from Phenomenex (Torrance, CA, USA). Gradient elution was performed at a flow rate of 0.4 ml min^−1^ at a column temperature of 40°C from 65% to 100% B within 7 min, followed by 100% B for 1.1 min and reconditioning at 65% for 3 min. The injection volume was 10 μl.

Electrospray ionisation (ESI)–tandem mass spectrometry (ESI-MS/MS) was performed using multiple reaction monitoring, and the reactions 267 to 147 for JH and 281 to 189 for JHEE were followed. The ESI source was operated in positive electrospray mode (ESI+) at 60°C, at a spray voltage of 3500 V. Argon was used as the collision gas. The capillary temperature was set at 300°C, with sheath gas pressure at 15 a.u. and auxiliary gas pressure at 15 a.u. The S-lens radio frequency amplitude was 50.93 for ion 267 and 47.76 for ion 281. The collision energy was 13 V for the reaction of ion 267 to yield the product ion 147, and 12 V for the reaction of ion 281 to yield the product ion 189. JH eluted out at 4.9 min and JHEE at 5.3 min. The calculated amounts obtained from MS measurements were multiplied by 4 (as 10 μl out of 40 μl was injected into the LC-MS/MS) and divided by the amount of haemolymph collected for each individual for the statistical analysis (raw data available at Unnikrishnan et al., 2024).

#### Gene expression measurements for *Vg*, *ilp-1* and transcription factors *usp*, *egr-1*, *BR-C* and *nautilus*

##### Brain dissection, RNA isolation, cDNA preparation and quantitative PCR

Collected worker samples were stored at −80°C until further processing. Honey bee brains were first dissected on a dry ice platform in a glass cavity block filled with 100% ethanol. The dissected brain and the corresponding abdomen of the bee were collected in individual tubes. The abdomen was used for measuring *Vg*, and brain was used for measuring *ilp-1* and transcription factors. The brain and the abdomen were then homogenised in TRIzol (Invitrogen, Life Technologies, Rockville, MD, USA) with a motorised homogeniser, followed by RNA extraction using the Trizol-chloroform method. Total RNA was extracted using Trizol (Invitrogen, Life Technologies) and chloroform and then precipitated using isopropyl alcohol mixed with ammonium acetate and glycogen. Glycogen (20 mg ml^−1^, Thermo Fisher Scientific, Life Technologies) was added to increase the recovery of RNA. Finally, samples were treated with DNase I (Invitrogen) to remove any possible DNA contamination. Extracted RNA concentration was measured using a NanoDrop spectrometer (Thermo Fisher Scientific). Approximately 800 ng of extracted RNA was converted to cDNA using Reverse Transcriptase SuperScript^TM^ III First-Strand enzyme and oligo(dT)_16_ primers (Invitrogen, Life Technologies) at 42°C for 60 min and 95°C for 5 min.

Quantitative PCR (qPCR) was performed on a mixture of 3 μl of 10×diluted cDNA (Millipore water), 5 μl Kapa SYBRGreen (Kapa Biosystems, Wilmington, MA, USA), and 1 μl forward and reverse primer (2.5 μmol). Reactions were run using a 7900HT Fast Real-Time PCR system (Applied Biosystems, Carlsbad, CA, USA) under the following conditions: 95°C for 5 min, 40 cycles of 95°C for 15 s, 60°C for 1 min, and 72°C for 30 s. Each biological replicate was analysed in a technical triplicate. For sample normalisation, the standard curve method was followed with *AcRP49* and *AfRP49* as the internal control for *A. cerana* and *A. florea*, respectively. We conducted a separate experiment testing six candidate reference genes (*Rp49*, *Rps18*, *Ef1-alpha*, *ElfS8*, *GAPDH* and *B-actin*) to identify a reference gene suitable for age-related studies in *A. florea* and *A. cerana*. For all six housekeeping genes, we determined brain and abdomen expression levels of bees ranging from 5 days to 50 days (further details are provided in Table S3; raw data are available at doi:10.6084/m9.figshare.14529129). Purity of all the qPCRs was verified using dissociation/melt curve analysis, and reactions with bad dissociation curves were discarded from the analysis. Although all primers generally showed efficiency of 95–100%, except the one for *AfUsp* (Table S2), standard curves with a separate stock cDNA were generated in each qPCR (384-well) plate to reduce inter-run variability. Test genes and *Rp49* levels in all samples from the same experiment were analysed in the same plate. Quantification of mRNA levels was based on the linear values interpolated from the standard curves. qPCR measurements and calculations are documented in Unnikrishnan et al. (2024).

Primers for *usp*, *egr-1*, *Amilp-1*, *Vg* and *Rp49* were designed based on *A. mellifera* primers used in previous literature (Table S1). The *A. mellifera* primer sequence was aligned on the respective *A. cerana* and *A. florea* genes using NCBI BLAST. After aligning the primers, differences in nucleotides, if any, were corrected such that the primer sequence matched 100% with the respective gene sequence. We newly designed primers for *AmNau* and *Br-C* because the published *A. mellifera* primers for *BR-C* showed poor efficiencies and we did not find any published data for *AmNau* primers (Table S1).

### Statistical analysis

All statistical analyses were performed in R version 3.4.1 (https://www.r-project.org/) with RStudio IDE (https://posit.co/).

#### Onset of foraging

For the onset of foraging, a linear mixed effects model with species (two levels, *A. florea* and *A. cerana*) and an interaction term between species and age as fixed effects, and the cumulative percentage of bees that initiated foraging as the response variable was built using the lme4 package (Bates et al., 2015) in R. The nests were the random effect.

#### Age-based comparison (JH and *Vg*)

For the age-based changes in JH levels, two separate mixed effects models for *A. florea* and *A. cerana* were built using the gamlss package in R (Rigby and Stasinopoulos, 2005). Age and foraging status (whether a bee were a forager or not) were the fixed effects, and the JH titres were the response variable, with colony and batch number (of using the MS machine) as the random effects fitted with a Weibull distribution. For the analysis of the temporal dynamics of *Vg* levels, separate mixed effects models were built for *A. florea* and *A. cerana*. In both cases, the fixed effect was age, and *Vg* gene expression levels was the response variable, with colony as the random effect. For *A. cerana*, a lognormal distribution was fit to the model using the lme4 package; for *A. florea*, a Weibull distribution was fit using gamlss package.

#### Nurse–forager comparison

For the comparisons of JH titres between nurses and foragers, separate mixed effects models were built for *A. florea* (fitted with a normal distribution) and *A. cerana* (fitted with lognormal distribution), with JH titres as the response variable, the behavioural state (nurse and forager) as the fixed effect, and colony as a random effect. For the gene expression comparisons (*Vg*, *ilp-1*, *usp*, *egr-1*, *BR-C* and *nautilus*) between nurses and foragers, the values were scaled to normalise for colony variation, and linear regression models were fit using the MASS package (Venables and Ripley, 2002). Separate models were built for each species and for each of the response variables, i.e. expression levels of *Vg*, *ilp-1* and the four transcription factors *usp*, *egr-1*, *BR-C* and *nautilus* – with the behavioural state, nurse and forager, as a categorical predictor variable. The correlations were analysed using Kendall's correlation coefficient, and ggplot2 (Wickham, 2016) was used for data visualisation. Dharma package (Hartig, 2022) was used to test model assumptions, and model comparisons were made using Akaike's information criterion values.

## RESULTS

### Onset of foraging in *A. florea* and *A. cerana*

**Fig. 1.**
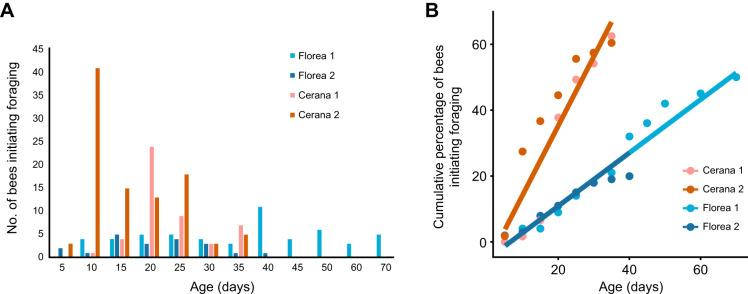
**Temporal dynamics of the onset of foraging in** ***Apis florea*** **and** ***Apis cerana*****.** (A) The number of bees initiating foraging on each day, in two colonies each of *A. florea* (shades of blue) and *A. cerana* (shades of red). (B) The onset of foraging in two colonies each of *A. florea* (shades of blue) and *A. cerana* (shades of red). The *y*-axis indicates the cumulative percentage of bees that initiated foraging out of the total number of marked bees released into the colony. A linear mixed effects model with species and interaction term between age and species as fixed effects, cumulative percentage as response variable and colony as random effect was built. The interaction term was significant for both species, indicating the difference in the speed of foraging initiation between the two species (Table S5).

We found that the age at the earliest initiation of foraging was similar in *A. florea* and *A. cerana*. However, the onset of foraging showed higher inter-individual variation in *A. florea* than in *A. cerana*. In colony Florea 1 of *A. florea*, by day 7, the first individual had initiated foraging, and, by day 70, 50% (50/100) of bees had become foragers. In the case of colony Florea 2 of *A. florea*, by day 4, the first foraging was initiated, and, by day 40, only 20% (20/100) of bees had become foragers (Fig. 1A,B). In colony Cerana 1 of *A. cerana*, first foraging was initiated at day 10, and, by day 35, 62% (48/77) of bees had become foragers (Fig. 1A,B); 35% (27/77) of the bees had disappeared before day 22 (see Fig. S1), and 2.5% (2/77) were still alive at day 24 and had not become foragers by day 35. In colony Cerana 2 of *A. cerana*, foraging was first observed on day 4, and, by day 35, 60% (98/164) of bees had become foragers (Fig. 1A,B); 36.5% (60/164) of the bees had disappeared before day 22 (see Fig. S2), and 3.6% (6/164) were still alive at day 24 but had not become foragers by day 35. The slope of the cumulative percentage of individuals initiating foraging was steeper for *A. cerana* than for *A. florea*. (Fig. 1A,B, Table S5). These percentages are from the total number of marked bees released into each colony. The percentages of bees that became foragers out of the total number of bees remaining in each colony are shown in Fig. S3.

### Temporal dynamics of JH and *Vg* expression

#### JH

**Fig. 2.**
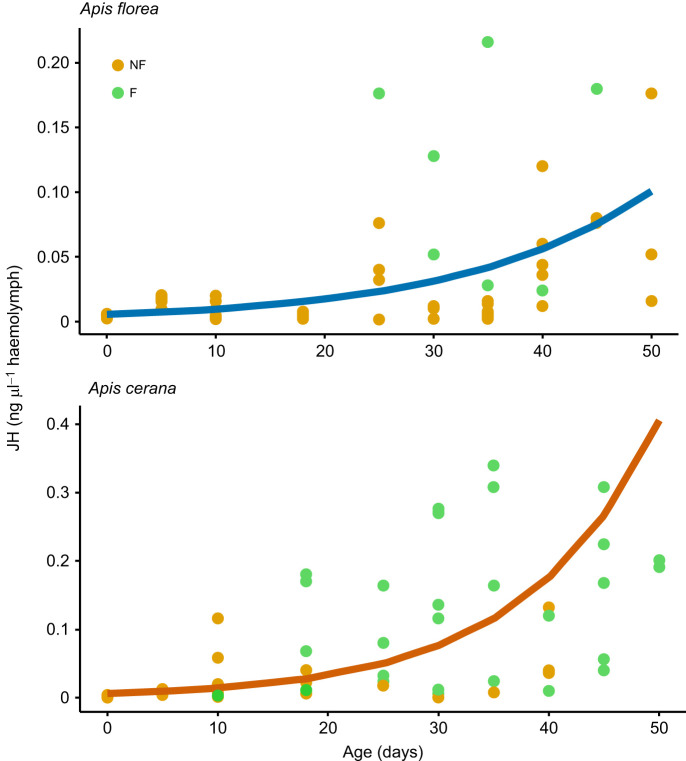
**Temporal dynamics of juvenile hormone (JH) titers in** ***A. florea*** **and** ***A. cerana*****.** Each dot indicates a single bee. F, bees that had become foragers by the time of collection; NF, bees that had not yet become foragers by the time of collection. Mixed effects models with age and foraging status as fixed effects, JH titres as response variable, and colony and batch number as random effects were built. Lines indicate the prediction from the models for the relationship between age and JH. In both species, age and forage status had a significant effect on JH levels (Table S6).

For both *A. florea* and *A. cerana*, age (*A. florea*: estimate, 0.055±0.006; CI=0.044–0.066; *P*<0.0001; *A. cerana*: estimate, 0.066±0.02; CI=0.03–0.1; *P*=0.0015) and foraging status (*A. florea*: estimate, 3.47±1.6; CI=0.37–6.6; *P*=0.03; *A. cerana*: estimate, 2.1±0.8; CI=0.53–3.6; *P*=0.01) had a statistically significant effect on the JH titres, although the estimate values were low for age. The interaction term between age and forage status was not significant in either species (*A. florea*: estimate, −0.054±0.045; CI=−0.14–0.03; *P*=0.24; *A. cerana*: estimate, −0.035±0.03; CI=−0.09–0.02; *P*=0.23) (Fig. 2, Table S6).

#### Vg

**Fig. 3.**
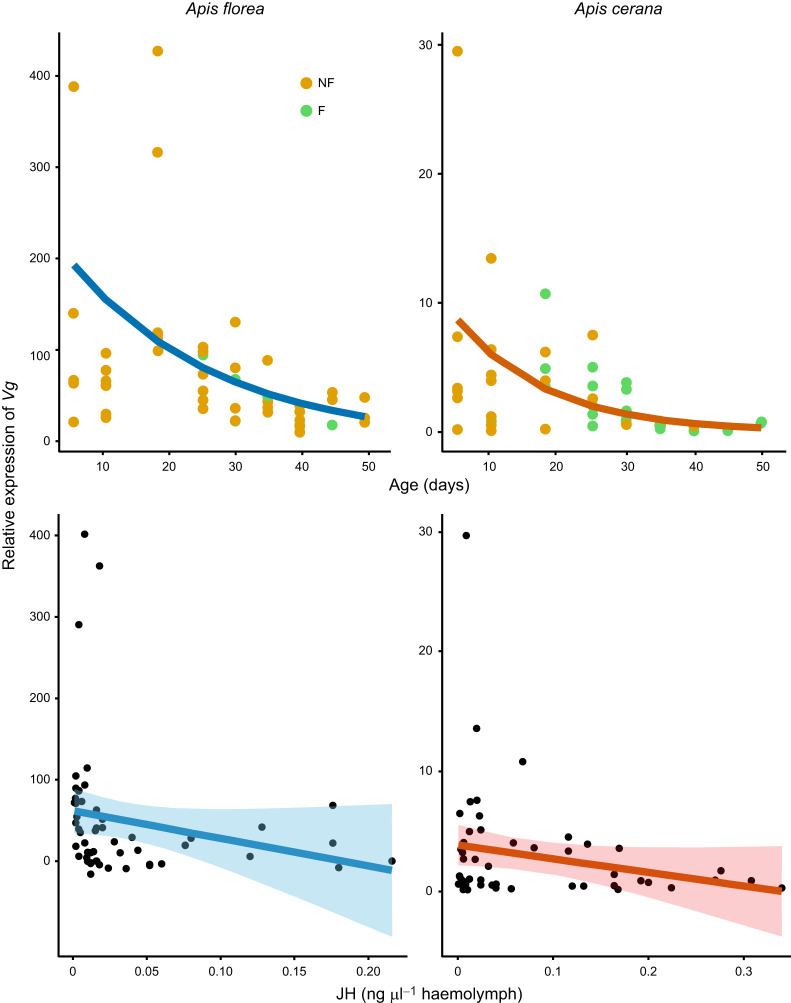
**Changes in vitellogenin (*Vg*) expression with age, and correlation between *Vg* and JH in *A. florea* and *A. cerana*.** Top row shows the changes in *Vg* expression with age; bottom row shows the correlation between *Vg* and JH. Each dot indicates a single bee. F, bees that had become foragers by the time of collection; NF, bees that had not yet become foragers by the time of collection. Mixed effects models with age as fixed effect, *Vg* levels as response variable and colony as random effect were built. Lines indicate the prediction from the models for the relationship between age and *Vg*. In both species, age did not have a significant effect on *Vg* levels, and there was no significant correlation between JH titres and *Vg* levels (Table S7).

There was no significant effect of age on *Vg* levels for both *A. florea* (estimate, −0.03±0.02; CI=−0.06–0.006; *P*=0.125) and *A. cerana* (estimate, −0.05±0.03; CI=−0.1–0.006; *P*=0.08) (Fig. 3, Table S7). Because JH and *Vg* were measured in the same bees, we checked for correlation between JH and *Vg*, but there was no significant correlation in either *A. florea* (coefficient, −0.136; CI=−0.46–0.22, *P*=0.449) or *A. cerana* (coefficient, −0.122; CI=−0.43-0.22, *P*=0.486) (Fig. 3).

### JH titres and expression of *Vg* and *ilp-1* in nurses and foragers

#### JH

**Fig. 4.**
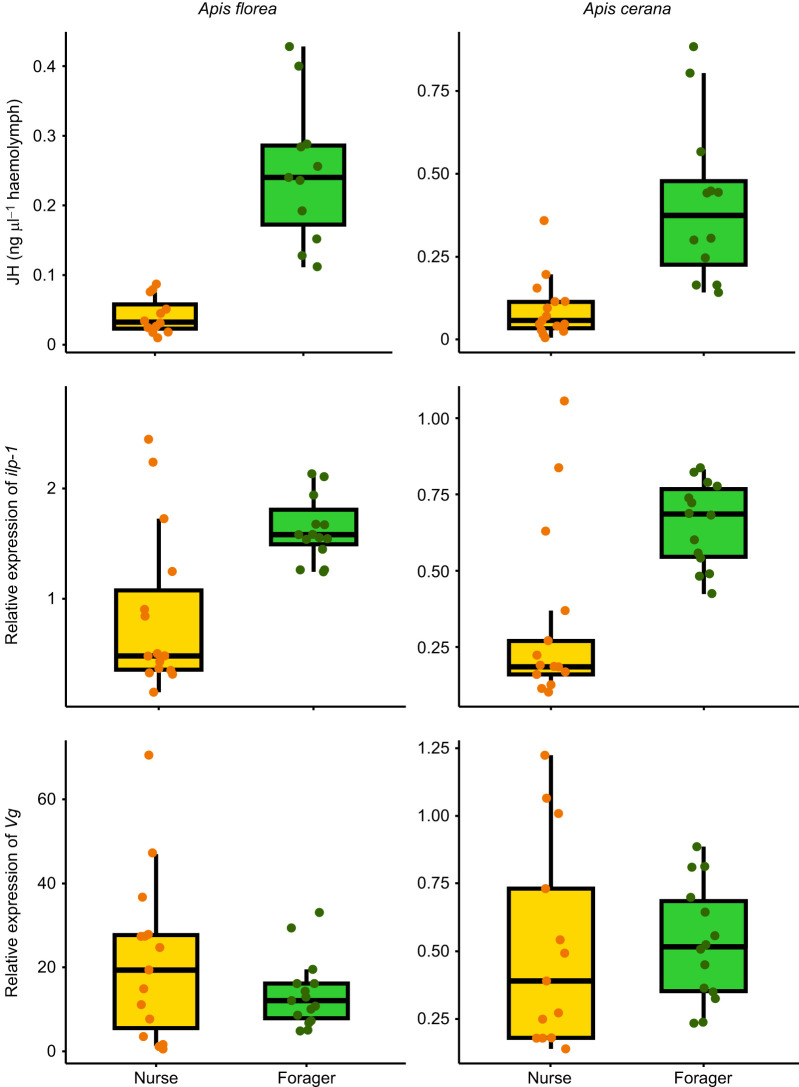
**Nurse–forager difference in JH titres and gene expression levels (*****Vg*****,** ***ilp-1*****) in** ***A. florea*** **and** ***A. cerana*****.** JH titres (top row), and insulin-like peptide (*ilp-1*; middle row) and *Vg* (bottom row) expression levels in nurses and foragers are shown for *A. florea* (left column) and *A. cerana* (right column). Results for colonies are pooled. Each dot indicates a single bee; orange indicates nurses and green indicates foragers. Boxes show the interquartile range (IQR) with medians; whiskers extend to 1.5×IQR, and points beyond are outliers. Regression models were built separately for each of the response variables (JH titres, *Vg* and *ilp-1* expression levels) and for each of the species. The predictor variable was behavioural state (categorical variable), i.e. nurse or forager. Although values were scaled for analysis for *Vg* and *ilp-1*, actual values are plotted in the figure. JH titres and *ilp-1* expression levels were significantly higher in foragers than in nurses for both species. *Vg* expression did not differ between nurses and foragers in both species (Tables S8–S10). Colonies are plotted separately in Fig. S4.

Linear mixed effects models (Table S6) showed significant differences between nurses and foragers in their JH titres, with foragers having significantly higher levels of JH than nurses for both *A. florea* (estimate, 0.21±0.03; CI=0.16–0.26; *P*<0.0001) and *A. cerana* (estimate, 1.6±0.5; CI=0.59–2.5; *P*=0.002) (Fig. 4, Table S8).

#### Vg

Linear regression models (Table S6) showed no significant differences in the levels of *Vg* between nurses and foragers for both *A. florea* (estimate, −0.49±0.35; CI=−1.2–0.2; *P*=0.17) and *A. cerana* (estimate, 0.11±0.38; CI=−0.7–0.9; *P*=0.77) (Fig. 4, Table S9).

#### ilp-1

Linear regression models showed that *ilp-1* was significantly higher in foragers than in nurses for both *A. florea* (estimate, 1.13±0.3; CI=0.54–1.72; *P*=0.0005) and *A. cerana* (estimate, 1.1±0.3; CI=0.5–1.68; *P*=0.0013) (Fig. 4, Table S10).

### Expression levels of brain transcription factors

**Fig. 5.**
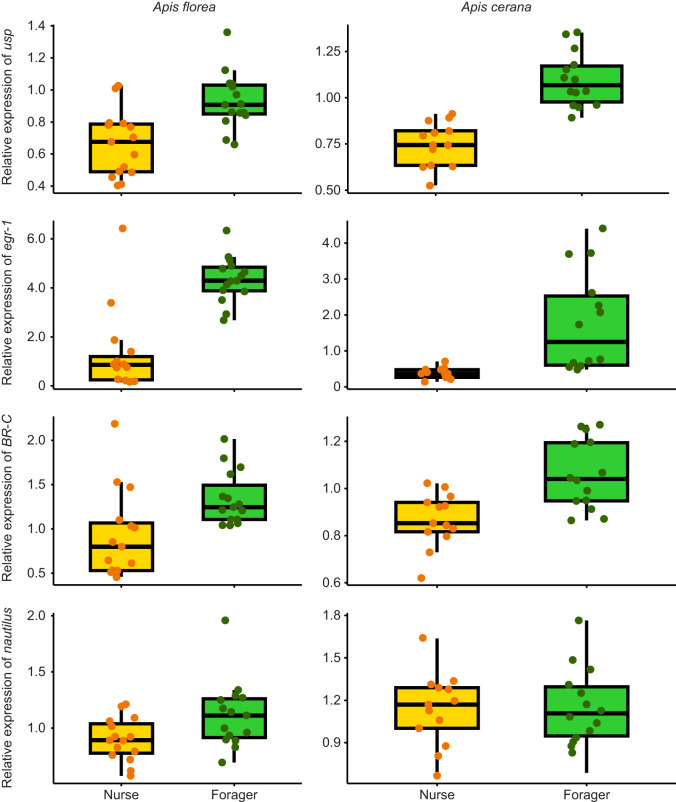
**Nurse-forager difference in expression of four major developmental transcription factors (*****usp*****,** ***egr-1*****,** ***BR-C*****,** ***nautilus*****) in** ***A. florea*** **and** ***A. cerana*****.** Expression levels of the transcription factors *usp*, *egr-1*, *BR-C* and *nautilus* are shown for three colonies of *A. florea* in the left column and for three colonies of *A. cerana* in the right column. Results for colonies are pooled. Dots indicate individual bee values for the transcription factors. Boxes show the IQR with medians; whiskers extend to 1.5×IQR, and points beyond are outliers. Linear regression models were built with each transcription factor as the response variable and behavioural state (nurse and forager) as predictor variable. Although values were scaled for analysis, actual values are plotted in the figure. Expression of all transcription factors except *nautilus* was significantly higher in foragers than in nurses for both species. *nautilus* expression did not differ between nurses and foragers in *A. florea* and *A. cerana* (Tables S11 and S12). Colonies are plotted separately in Fig. S5.

All the transcription factors measured, i.e. *usp* (*A. florea*: estimate, 1.08±0.29; CI=0.5–1.7; *P*=0.001; *A. cerana*: estimate, 1.03±0.3; CI=0.4–1.7; *P*=0.003), *egr-1* (*A. florea*: estimate, 1.5±0.2; CI=1.06–1.92; *P*<0.0001; *A. cerana*: estimate, 1.44±0.24; CI=0.95–1.93; *P*<0.0001) and *BR-C* (*A. florea*: estimate, 0.92±0.31; CI=0.27–1.56; *P*=0.007; *A. cerana*: estimate, 0.8±0.34; CI=0.11–1.5; *P*=0.02), except for *nautilus*, were significantly more highly expressed in brains of foragers than in brains of nurses for both *A. florea* (Fig. 5, Table S11) and *A. cerana* (Fig. 5, Table S12). *nautilus* (*A. florea*: estimate, 0.64±0.34; CI=−0.06–1.33; *P*=0.07; *A. cerana*: estimate, 0.63±0.35; CI=−0.09–1.35; *P*=0.08) was not significantly different between nurses and foragers in *A. florea* and *A. cerana* (Fig. 5, Tables S11 and S12).

## DISCUSSION

Our study provides four general findings. First, *A. cerana* workers show an accelerated behavioural maturation compared with that of *A. florea* workers. Second, all foraging-promoting hormonal and molecular traits (JH, *ilp-1*, and transcription factors *usp* and *egr-1*) were similar between the workers of both species and mirrored those reported in *A. mellifera*. Third, expression patterns of nurse status-associated regulators (*Vg* and transcription factors *nautilus* and *BR-C*) were similar between workers of both species but differed from expression patterns in *A. mellifera* workers. Fourth, *A. florea* workers showed substantially higher levels of *Vg* expression than did the *A. cerana* workers.

The results of our behavioural experiments indicate that *A. florea* and *A. cerana* workers are competent to become foragers within a few days after emergence but markedly differ in their developmental pace. At the population level, *A. cerana* workers show an accelerated behavioural maturation compared with that of *A. florea* workers. However, this faster development is not based on general acceleration of the behavioural maturation but on reduction of the individual variation in developmental pace. Such a decrease in developmental variation could be due to a tighter regulation of JH titres and dynamics. As the colonies of both species were examined in parallel during flowering seasons and the colonies were of similar size (visual evaluation), we are confident that the detected differences in behavioural maturation present actual species differences. Our results are also corroborated by the findings of earlier studies in which the two species were independently studied (Bhagavan and Brockmann, 2019; Huang et al., 2001; Rahman et al., 2025). However, to which extent worker behavioural maturation in *A. cerana* and *A. florea* show some degrees of plasticity with respect to flowering season or colony status, as has been shown in *A. mellifera* (Amdam et al., 2005; Fluri et al., 1982; Seeley and Visscher, 1985), remains unanswered. As *A. florea* (open nesting) is phylogenetically ancestral, our findings suggest that the accelerated behavioural maturation in the cavity-nesting species, *A. cerana* and *A. mellifera*, is a derived trait (Lo et al., 2010; Raffiudin and Crozier, 2007). A preliminary report suggests that *A. dorsata* workers also show a slow behavioural maturation, but detailed formal experiments are missing (Otis et al., 1990).

A probable hypothesis regarding ultimate causes of the differences in the pace of behavioural development between open-nesting versus cavity-nesting bees is based on the observation that the brood-to-worker ratio is higher in cavity-nesting species than in open-nesting species, which is consistent with a higher worker production rate (Dyer and Seeley, 1991; Huang et al., 2001; Oldroyd and Wongsiri, 2006). In addition, abandoning the curtain in cavity-nesting species likely allowed more workers to be engaged in nursing and foraging and may also have facilitated a higher worker production rate. This higher worker production rate, in turn, allows a faster colony growth and a higher capacity to replace foragers, which are exposed to higher mortality risks (Neukirch, 1982; Page and Peng, 2001; Rueppell et al., 2007; Visscher and Dukas, 1997). Thus, cavity nesting facilitates faster replacement and, consequently, earlier availability of new foragers. Conversely, open nesting results in constraints due to lower brood–worker ratio as well as the need to maintain a curtain of bees at all times required for protection and thermoregulation. The more distributed onset of foraging observed in this study might, therefore, be a strategy to generate a buffer against detrimental losses in the forager population (Seeley et al., 1982; Young et al., 2021).

Similar to findings in *A. mellifera*, we found a similar age-related increase in JH titres in *A. cerana* and *A. florea* (Fig. 2), suggesting that the age-linked increase in JH is a conserved feature across honey bee species. However, this relationship may be partly influenced by behavioural state, particularly in the case of *A. cerana*, in which several of the sampled bees had already begun foraging, raising the possibility that the decision to initiate foraging itself could contribute to elevated JH levels. Supporting this idea, we also observed clear behavioural correlates of JH: when we examined behaviourally identified nurse bees and foragers, JH titres were significantly higher in foragers than in nurses in both species. This finding mirrors the well-documented role of JH in *A. mellifera* (Elekonich et al., 2001; Huang et al., 1994; Jassim et al., 2000) and is further corroborated by previous studies in *A. cerana* (Diao et al., 2018; Huang et al., 2001). Further, corresponding with the JH dynamics, we found, in both species, an increase in *ilp-1* expression as well as robust upregulation of the foraging-associated transcriptions factors *usp* and *egr-1* in foragers compared with nurses as reported for *A. mellifera* (Ament et al., 2008, 2012; Chandrasekaran et al., 2011; Khamis et al., 2015). Together, these findings suggest that major foraging-facilitating hormonal and transcriptional regulatory mechanisms are likely to be conserved across honey bee species.

In contrast to the consistent patterns we observed with JH, *Vg* expression levels showed no significant association with either age or behavioural role in *A. cerana* and *A. florea* (Fig. 3), and there was no significant correlation between JH and *Vg* levels in either species. In *A. mellifera*, Vg is typically elevated in nurses and declines with age, and experimental knockdown of *Vg* expression led to precocious foraging (Amdam et al., 2003; Engels, 1974; Ihle et al., 2010; Marco Antonio et al., 2008; Nelson et al., 2007). A similar nurse-biased expression pattern has also been reported in *A. cerana* populations from subtropical Central China (Diao et al., 2018 supplementary material). However, Vg titres differ between temperate and tropical populations of *A. mellifera*, and *Vg* expression levels are dependent on the presence of brood and young workers (Amdam et al., 2005; Eyer et al., 2017). In contrast to JH, which was measured as circulating hormone titres, *Vg* was quantified here at the level of gene expression rather than haemolymph Vg protein titres. Although abdominal *Vg* mRNA levels have been widely used to examine age- and task-related variation (Corona et al., 2007; Eyer et al., 2017; Klett et al., 2025), transcriptional and circulating protein levels may not always be tightly coupled. For example, as Vg is turned over to brood food, Vg protein levels might be low in nurse bees, whereas the mRNA levels might remain high or even increase (Amdam et al., 2005). The same authors also suggested that Vg protein levels are homeostatically regulated, i.e. in the case of high Vg protein content, *Vg* mRNA levels could be downregulated (Amdam and Omholt, 2003). Thus, future studies measuring Vg titres and *Vg* mRNA expression in parallel might reveal more specific differences in Vg function and signalling between honey bee species and populations.

The clearest result of our *Vg* measurements is the much higher *Vg* expression in *A. florea* workers than in *A. cerana* workers, which we consistently detected in all four experiments. Although this result was very surprising to us, Engels (1974) reported observing exceptionally high Vg protein concentration in the haemolymph of *A. florea* workers unique among honey bee species. Thus, both datasets strongly suggest that workers of *A. florea* – and maybe those of the sister species *Apis andreniformis* – are likely to be unique in the capacity to synthesise Vg. The function of this is unclear, but it appears not to be related to reproductive potential as workers do not show any significant level of ovary activation (Halling et al., 2001). An alternative hypothesis might be that the slower-developing workers also function as additional food storage units, transforming pollen into storage proteins. Recently, it was shown that 10- and 14-day-old *A. mellifera* workers show higher *Vg* expression levels during swarm preparation (Klett et al., 2025), suggesting that accumulated Vg stores could be used as a colony food store during a period of reduced foraging activity (Amdam et al., 2005; Klett et al., 2025).

Similarly to *Vg*, the expression patterns of the two transcription factors linked to nursing behaviour, *BR-C* and *nautilus*, also diverged from those reported in *A. mellifera* (Hamilton et al., 2019; Khamis et al., 2015). Rather than being upregulated in nurses, *BR-C* expression was unexpectedly higher in foragers, and *nautilus* expression did not differ significantly between the behavioural castes. These results are intriguing as, for example, functional studies using RNA interference against *BR-C* have revealed effects on brood-related behaviours and on the regulation of behavioural maturation, including transitions linked to foraging onset in *A. mellifera* (Hamilton et al., 2017, 2019). The lack of a clear nurse-specific upregulation for *BR-C* and *nautilus* in both *A. florea* and *A. cerana* may reflect species-specific differences in the molecular mechanisms underlying division of labour, possibly due to evolutionary divergence or ecological adaptation. However, we think that the similar expression patterns for *Vg*, *BR-C* and *nautilus* in *A. florea* and *A. cerana* suggest important differences between tropical and temperate honey bee lineages in nurse-associated hormonal and transcriptional regulation. In this respect, comparative studies of behavioural maturation in tropical and temperate *A. mellifera* and *A. cerana* populations will be very interesting.

To summarise, our study demonstrates that worker behavioural maturation is slow in the most ancestral recent honey bee species and accelerated in the cavity-nesting honey bees. Although it is tempting to hypothesise that this difference in behavioural pace is associated with the nesting behaviour, we still do not know anything about worker adult development in giant honey bees or even the sister species of *A. florea*. The molecular results of our study suggest that the foraging-facilitating hormonal and transcriptional factor dynamics are conserved among the three, so far, studied honey bee species, whereas the nurse status-associated hormonal and transcriptional factor dynamics might show important variation. We propose that, in addition to the evolutionary change in nesting behaviour, expansion of the distribution range of *A. mellifera* and *A. cerana* populations into temperate zones and the evolution of long-lived winter bees might have led to changes in worker behavioural maturation and associated molecular mechanisms. Workers of the tropical-adapted Africanised *A. mellifera* population show some plasticity in longevity during the wet season, but long-lived worker bees, such as the winter bees of the temperate populations, have not yet been reported (Feliciano-Cardona et al., 2020). Also, the temperate and subtropical populations of *A. cerana* likely should have evolved long-lived winter bees, but this has not yet been demonstrated.

Our understanding of the biology and ecology of honey bees and that of the European honey bees, *Apis mellifera carnica* and *Apis mellifera ligustica*, which are the workhorses of industrialised agriculture worldwide, will benefit from comparative studies including Asian honey bee species. However, molecular studies on Asian honey bees with reference to our knowledge on *A. mellifera* might be a fruitful shortcut to identifying physiological differences that hint at differences in the biology and behaviour of the species. These findings then could guide more time-intensive behavioural observations and experiments. Given the importance of the Asian honey bees for pollination services in tropical Asia, basic and applied studies on the biology and ecology of these species are urgently needed (Warrit et al., 2023).

## Supplementary Material

10.1242/jexbio.251399_sup1 Supplementary information
