# Supplementary material for: Variation in behavioural maturation in tropical honey bees corresponds with hormonal and molecular differences
Source: J Exp Biol. 2026 Apr 23;229(8):jeb251399. doi: 10.1242/jeb.251399 (PMC13143204; doi:10.1242/jeb.251399)
Supplement: Supplementary information [file jexbio-229-251399-s1.pdf]

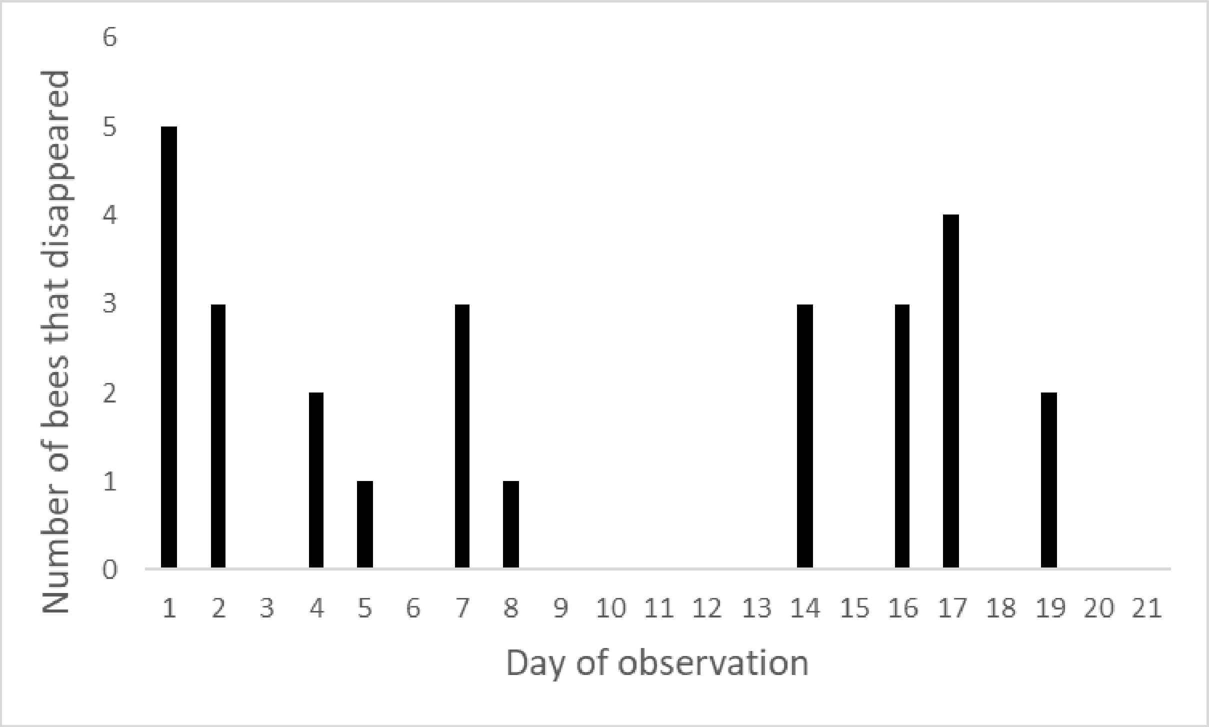

Fig. S1. Individuals that disappeared on each day (the day they were last seen) during observations in the colony, Cerana 1.

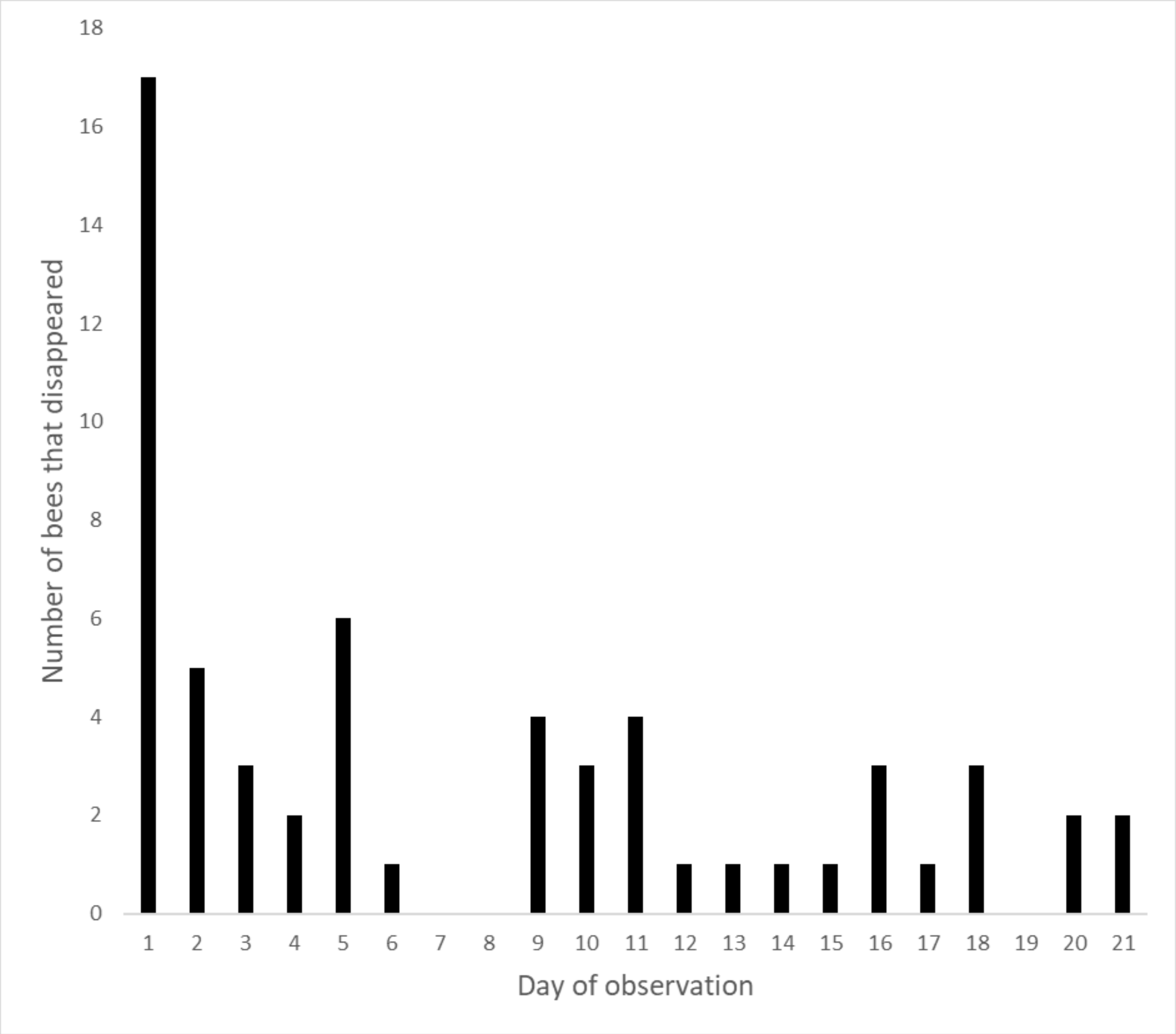

Fig. S2. Individuals that disappeared on each day (the day they were last seen) during observations in the colony, Cerana 2.

Journal of Experimental Biology • Supplementary information

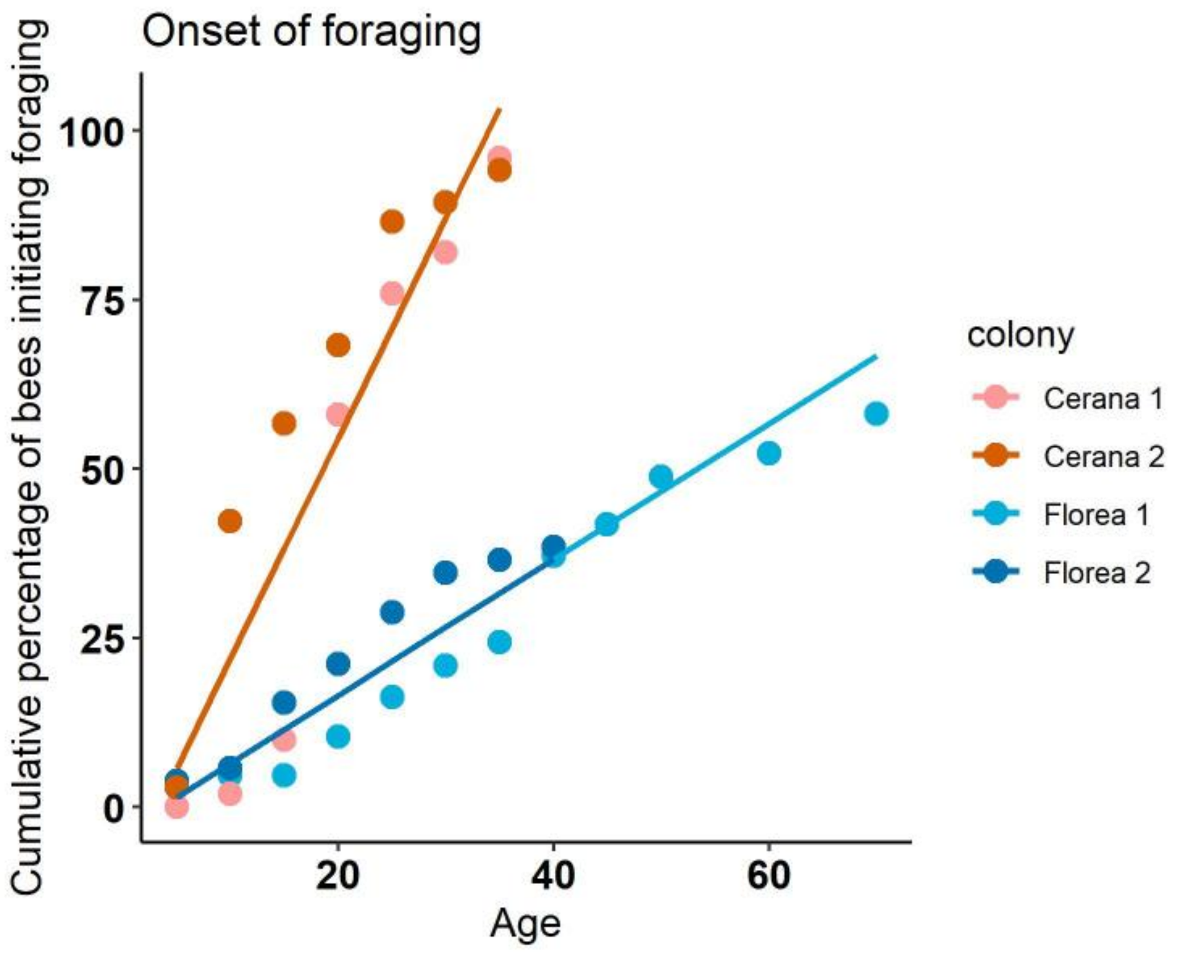

Fig. S3. Cumulative percentage of bees that became foragers out of the number of bees that were still alive (which was 50 bees for Cerana 1 and 104 bees for Cerana 2) by the end of the observations in the case of *A. cerana* . 48 bees out of 50 remaining bees (96%) had become foragers in Cerana 1 and 98 bees out of 104 remaining bees (94%) had become foragers in Cerana 2. In the case of *A. florea* since observations were not possible on the comb the exact age/day of disappearance of bees was difficult to calculate and so cumulative percentage of bees that became foragers out of the total number of bees that were spotted on the curtain (which was 86 for Florea 1 and 52 for Florea 2) during observations was calculated. 50 bees out of 86 bees (58%) spotted on curtain became foragers in Florea 1 and 20 bees out of 52 bees (38%) spotted on curtain became foragers in Florea 2.

Journal of Experimental Biology • Supplementary information

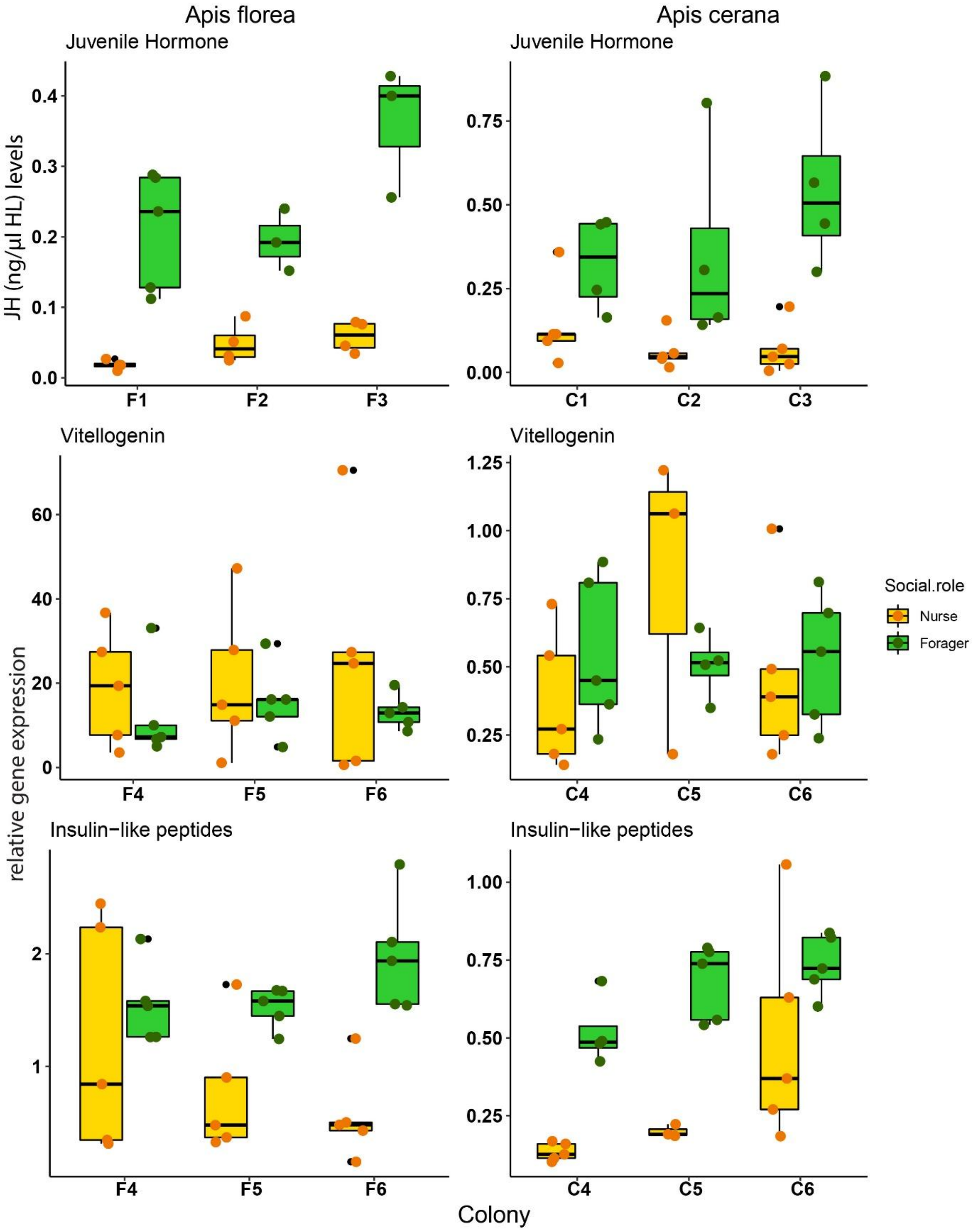

Fig. S4. Indicating the levels of JH, Vg, ilp-1 comparing nurses and foragers for *A. florea* and *A. cerana*, with the colonies plotted separately.

Journal of Experimental Biology • Supplementary information

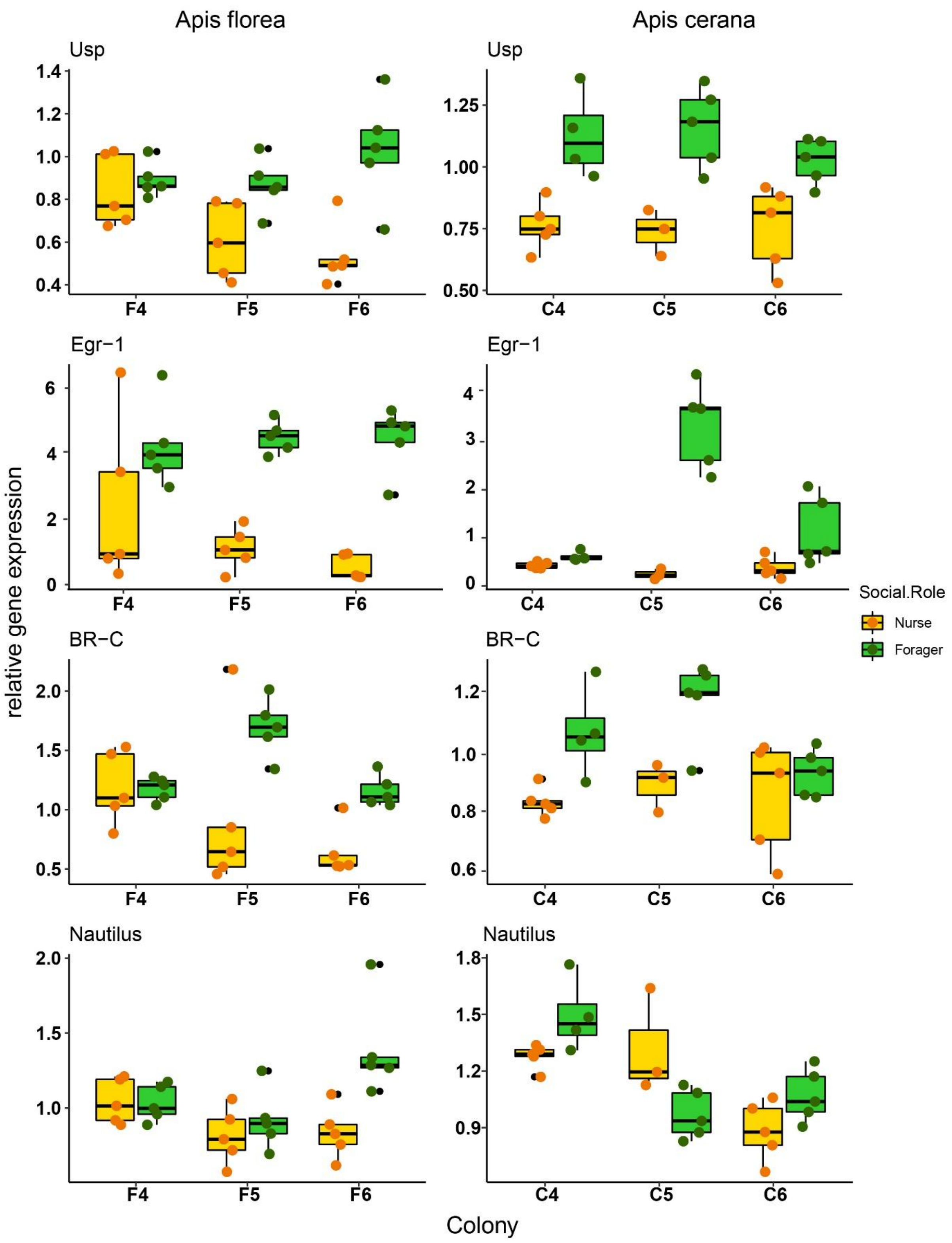

Fig. S5. Figure showing TF levels comparing between nurses and foragers for *A. florea* and *A. cerana*, with colonies plotted separately.

Journal of Experimental Biology • Supplementary information

Table S1. Primers for the transcription factors, *Vg, Ilp-1-1* and *Rp49*.

Gene Name	Species	NCBI Reference Sequence	Oligonucleotide primer sequence 5'-3'	Size (bp)	Reference *A. mellifera* Primer obtained from
Acusp	*A. cerana*	XM_017066341.2	F-TTGGCTAAGTCTGGACAAC<br>R- TAGGGTGCGACTGCTTTG	207	Singh et al.,2018
AcEgr-1	*A. cerana*	NW_016019075.1	F-GCTCTGAGGGTGATTTCTCG<br>R-GAGAAACCGTTCTGCTGTGA	138	Singh et al.,2018
AcIlp-1-1-1	*A. cerana*	XM_028666747.1	F-GCTCAGGCTGTGCTCGAAAAGT<br>R-CGTTGTATCCACGACCCTTGC	68	Corona et al., 2007
AcNau	*A. cerana*	XM_017057127.2	F-TCGCAACCATTACGATACGC<br>R-TAAACATCGGCGAGGTCCAT	239	NA

Journal of Experimental Biology • Supplementary information

AcBR-C	***A. cerana***	**XM_017058225.2**	**F-GCTCAACAACAACGACGCTA**<br>**R-TTACCGCTGTTACCACCTGT**	**162**	**NA**
AcVg	***A. cerana***	**NM_001328484.1**	**F- CAAGTTCCGACCGACAAC**<br>**R-ATCACGAAGTCCGACAAAG**	**108**	**Corona et al., 2007**
AcRP49	***A. cerana***	**XM_017056470.2**	**F-CGTCACATGTTGCCAACTGGT**<br>**R-TGAGCACGTTCAACAATGG**	**150**	**Singh et al.,2018**
Afusp	***A. florea***	**XM_012492027.2**	**F-TTGGCTAAGTCTGGACAAC**<br>**R- TAGGGTGCGACTGCTTTG**	**207**	**Singh et al.,2018**
AfEgr-1	***A. florea***	**XM_012489705.2**	**F-GAGAAGCCGTTCTGCTGTGA**<br>**R- GCTCTGGGGGTGATTTCT**	**138**	**Singh et al.,2018**
AfIlp-1-1-1	***A. florea***	**XM_003691440.3**	**F-GCTCAGGCTGTGCTCGAAAAGT**<br>**R-CGTTGTATCCACGACCCTTGC**	**168**	**Corona et al., 2007**
AfNau	***A. florea***	**XM_003697989.3**	**F-TCACAACCATTACGATACGC**<br>**R- TAAACATCGGCGAGGTCC**	**240**	**NA**

Journal of Experimental Biology • Supplementary information

AfBR-C	***A. florea***	**XM_031917427.1**	**F-TGAAGAACAACCACGTGTCG**<br><br>**R-TTACCGCTGTTACCACCTGT**	**118**	**NA**
AfVg	***A. florea***	**XM_003689645.3**	**F- AGTTCCGACTGACGACG**<br><br>**R- GTCCATCGCCCTTCAAC**	**158**	**Corona et al., 2007**
AfRP49	***A. florea***	**XM_012493364.2**	**F-CGTCACATGTTGCCAACTGGT**<br><br>**R-TTGAGCACGTTCAACAATGG**	**150**	**Singh et al.,2018**

Table S2. Primer efficiencies

Primer efficiency *Vg* age (*A. florea* & *A. cerana*)

Species	qPCR run	*Vg*		*Rp49*
Apis florea	Samples 5-30 days	101.9		98
	Samples 35-50 days	95.1		91.8
Apis cerana	Samples 5-30 days	103.6		85.6
	Samples 35-50 days	97.8		96.4

Primer efficiencies *Vg* nurse forager comparison (*A. florea* &*A. cerana*)

Species	*Vg*	*Rp49*
Apis florea	105.1	98.4
Apis cerana	89.4	96.4

Primer efficiencies transcription factors and *ilp-1* (*Apis florea*)

qPCR runs	Primer efficiencies					
	usp	*egr-1*	*Ilp-1*	*nau*	*BR-C*	*Rp49*
Colony F4	87.8	94.1	89.4	98.0	91.9	93.3
Colony F5	86.5	96.3	99.5	96.8		92.6
Colony F6	89.9	96.3	95.1	104.4	95.1	96.7

Primer efficiencies transcription factors and *ilp-1* (*Apis cerana*)

qPCR runs	Primer efficiencies					
	usp	*egr-1*	*Ilp-1*	*nau*	*BR-C*	*Rp49*
Colony 4	99.8	105.9	100.9	99.6		100.4

Colony 5	93.2	100.6	98.6	100.3		98.7
Colony 6	83.6	88.4	93.6	92.6		91.0
All colonies BrC					96.2	100.8

Suitability of reference gene

The bees collected for the study of age-related changes in JH were also used for the reference gene determining study as well. Brain and abdomen dissections, RNA isolation, cDNA preparation and quantitative PCR were done following the same protocol as mentioned above. A total of 10 qPCR plates were run with triplicate replicates for a total of 72 bees, ranging from ages 5 days to 50 days. To compare the different RNA transcription levels, the Ct values were directly compared and their variance was calculated as well. The stability and suitability of the reference genes were analysed using RefFinder, which incorporated 4 methods, Delta Ct, BestKeeper, NormFinder and Genorm and provided a comprehensive ranking based on these 4 methods to indicate reference genes in the order of most to least suitable. RefFinder suggested Rp49 as the most suitable reference gene for brain tissues and second best reference gene for abdomen tissues for both *A. florea* (Table S1 and S2) and *A. cerana* (Table S3 and S4).

Table S3. Results from RefFinder. Ranking Order (Better--Good--Average)

Apis florea brain					
**Method**	**1**	**2**	**3**	**4**	**5**
**Delta Ct**	Af_Rp49	Af_ElfS8	Af_Rps18	Af_Ef1alpha	Af_Gapdh
**BestKeeper**	Af_Rps18	Af_Rp49	Af_ElfS8	Af_Gapdh	Af_Ef1alpha
**Normfinder**	Af_ElfS18	Af_Rp49	Af_Rps18	Af_Ef1alpha	Af_Gapdh
**Genorm**	Af_Rp49<br>Af_Rps18		Af_ElfS8	Af_Ef1alpha	Af_Gapdh
**Recommended comprehensive ranking**	Af_Rp49	Af_Rps18	Af_ElfS8	Af_Ef1alpha	Af_Gapdh
A. florea abdomen					
**Method**	**1**	**2**	**3**	**4**	**5**
**Delta Ct**	Af_Gapdh	Af_ElfS8	Af_Rp49	Af_Rps18	Af_Ef1alpha
**BestKeeper**	Af_Rp49	Af_Rps18	Af_Gapdh	Af_ElfS8	Af_Ef1alpha
**Normfinder**	Af_Gapdh	Af_ElfS8	Af_Rp49	Af_Rps18	Af_Ef1alpha

**Genorm**	Af_Rp49<br>Af_Rps18		Af_Gapdh	Af_ElfS8	Af_Ef1alpha
**Recommended comprehensive ranking**	Af_Gapdh	Af_Rp49	Af_Rps18	Af_ElfS8	Af_Ef1alpha
Apis cerana brain					
**Method**	**1**	**2**	**3**	**4**	**5**
**Delta Ct**	Ac_Rp49	Ac_ElfS8	Ac_Betaactin	Ac_Rps18	Ac_Ef1alpha
**BestKeeper**	Ac_Rps18	Ac_Rp49	Ac_Betaactin	Ac_ElfS8	Ac_Ef1alpha
**Normfinder**	Ac_ElfS8	Ac_Rp49	Ac_Rps18	Ac_Betaactin	Ac_Ef1alpha
**Genorm**	Ac_Rp49<br>Ac_Betaactin		Ac_Rps18	Ac_ElfS8	Ac_Ef1alpha
**Recommended Comprehensive ranking**	Ac_Rp49	Ac_ElfS8	Ac_Betaactin	Ac_Rps18	Ac_Ef1alpha
A. cerana abdomen					
**Method**	**1**	**2**	**3**	**4**	**5**
**Delta_Ct**	Ac_ElfS8	Ac_Betaactin	Ac_Rp49	Ac_Rps18	Ac_Ef1alpha
**BestKeeper**	Ac_Rp49	Ac_Rps18	Ac_Betaactin	Ac_ElfS8	Ac_Ef1alpha
**Normfinder**	Ac_ElfS8	Ac_Rps18	Ac_Betaactin	Ac_Rp49	Ac_Ef1alpha
**Genorm**	Ac_Rp49<br>Ac_Betaactin		Ac_ElfS8	Ac_Rps18	Ac_Ef1alpha
**Recommended Comprehensive ranking**	Ac_ElfS8	Ac_Rp49	Ac_Betaactin	Ac_Rps18	Ac_Ef1alpha

Table S4. Details of colonies used for experiments

**Species**	**Colony ID**	**Experiment period**	**Colony size**	**Colony location**
Behavioral experiments – onset of foraging				

Apis cerana	Cerana 1	December 2017 – February 2018	4 frames	Bengaluru, India
	Cerana 2	April – June 2019	4 frames	Bengaluru, India
Apis florea	Florea 1	December 2018 – February 2019	N/A	Bengaluru, India
	Florea 2	October – November 2018	N/A	Bengaluru, India
Temporal dynamics of JH and Vg (also noted onset of foraging)				
Apis cerana	Cj1	November 2019 – January 2020	6 frames	Bengaluru, India
	Cj2	November 2019 – January 2020	6 frames	Bengaluru, India
Apis florea	Fj1	November 2019 – January 2020	N/A	Bengaluru, India
	Fj2	November 2019 – January 2020	N/A	Bengaluru, India
Nurse-Forager comparison - JH titers				
Apis cerana	C1	January 2021	5 frames	Bengaluru, India
	C2	January 2021	5 frames	Bengaluru, India
	C3	January 2021	5 frames	Bengaluru, India
Apis florea	F1	January 2021	N/A	Bengaluru, India
	F2	January 2021	N/A	Bengaluru, India
	F2	January 2021	N/A	Bengaluru, India
Nurse-Forager comparison - Vg, Ilp-1-1, TFs				
Apis cerana	C4	January 2021	5 frames	Bengaluru, India
	C5	January 2021	5 frames	Bengaluru, India
	C6	January 2021	5 frames	Bengaluru, India
Apis florea	F4	January 2021	N/A	Bengaluru, India
	F5	January 2021	N/A	Bengaluru, India
	F6	January 2021	N/A	Bengaluru, India

Table S5. Table showing the fixed effects, random effects and the estimate with standard error, confidence intervals and P values for the linear mixed effects model studying the onset of foraging behaviour in *A. florea* and *A. cerana.* The interaction term between age (in days) and species was significant for both species.

**Fixed effects**	**Estimate**	**Std. Error**	**CI**	**P value**
(Intercept)	-6.69	3.89	-16.2 – 2.8	0.103
speciesFlorea	1.47	5.04	-9.89 – 15.99	0.775
age:Cerana	2.102	0.15	1.8 – 2.4	< 0.0001****
age:Florea	0.808	0.07	0.85 – 1.15	< 0.0001****
**Random effects**	**Variance**			
Nest	8.79			

Journal of Experimental Biology • Supplementary information

Residual	30.13			

Table S6. Table showing the fixed effects, random effects and the estimate with standard error, confidence intervals and P values for the generalised linear mixed effects models (fitted with Weibull distribution) to study the changes in JH titres in haemolymph with age and forage status (whether a bee is a forager or not) for (a) *A. florea* and (b) *A. cerana*. Age and forage status were significant predictors for JH titres in both *A. florea* and *A. cerana*.

(a). *Apis florea*

**Fixed effects**	**Estimate**	**Std. Error**	**CI**	**P value**
(Intercept)	-5.52	0.153	-5.82 – -5.22	<0.0001****
age	0.055	0.0056	0.044– 0.066	<0.0001****
forage.st.F	3.47	1.58	0.37 – 6.57	0.033*
age:forage.st.F	-0.054	0.045	-0.14 – 0.03	0.238
**Random effects**	**Std.Dev**			
Nest	0.22			
Batch	0.56			
Residual	1.02			

(b). *Apis cerana*

**Fixed effects**	**Estimate**	**Std. Error**	**CI**	**P value**
(Intercept)	-5.19	0.31	-5.81 – -4.58	<0.0001****
age	0.066	0.019	0.03 – 0.1	0.0015**
forage.st.F	2.06	0.78	0.53 – 3.6	0.011*
age:forage.st.F	-0.035	0.03	-0.09 – 0.02	0.23
**Random effects**	**Std.Dev**			
Nest	2.81e-05			
Batch	0.0001			
Residual	1.22			

Table S7. Table showing the fixed effects, random effects and the estimate with standard error, confidence intervals and P values for the generalised linear mixed effects models (fitted with Weibull distribution) to study the changes in *Vg* gene expression levels in abdomen with age for *A. florea* and *A. cerana*. Age was not a significant predictor of *Vg* levels in both *A. florea* and *A. cerana*

(a) *A. florea*

Journal of Experimental Biology • Supplementary information

Fixed effects	Estimate	Std. Error	CI	P value
(Intercept)	5.18	0.35	4.49 – 5.87	<0.0001****
age	-0.027	0.017	-0.06 – 0.006	0.125
**Random effects**	**Std.Dev**			
Nest	1.61e-05			
Residual	1.23			

(b) *A. cerana*

Fixed effects	Estimate	Std. Error	CI	P value
(Intercept)	2.05	0.39	1.28 – 2.82	<0.0001****
age	-0.049	0.028	-0.1 – 0.006	0.083
**Random effects**	**Variance**			
Nest	3.1			
Residual	23.41			

Table S8. Table showing the fixed effects, random effects and the estimate with standard error, confidence intervals and P values for the linear mixed effects models to study JH titres in nurses and foragers in *A. florea*. Foragers had significantly higher titre levels of JH than nurses in both *A. florea* and *A. cerana*

(a) *A. florea*

Fixed effects	Estimate	Std. Error	CI	P value
(Intercept)	0.042	0.026	-0.025 – 0.11	0.17
Social.Role.F	0.209	0.026	0.156 – 0.262	<0.0001****
**Random effects**	**Variance**			
Nest	0.0011			
Residual	0.0038			

(b) *A. cerana*

Fixed effects	Estimate	Std. Error	CI	P value
(Intercept)	-2.47	0.511	-3.47 – -1.47	<0.0001****
Social.Role.F	1.56	0.49	0.59 – 2.53	0.0016**
**Random effects**	**Variance**			
Nest	0.024			
Residual	0.027			

Table S9. Table showing the predictor variable and the estimate with standard error, confidence intervals and P values for linear regression models to study *Vg* expression levels in nurses and foragers in *A. florea*. There was no significant difference between foragers and nurses in both *A. florea* and *A. cerana*.

(a) *A. florea*

**Predictor**	**Estimate**	**Std. Error**	**CI**	**P value**
(Intercept)	0.243	0.24	-0.26 – 0.74	0.33
Social.Role.F	-0.486	0.35	-1.19 – 0.22	0.17
**R^2**	**F-statistic**			
0.065	1.96			

(b) *A. cerana*

**Predictor**	**Estimate**	**Std. Error**	**CI**	**P value**
(Intercept)	-0.058	0.27	-0.62 – 0.5	0.834
Social.Role.F	0.111	0.38	-0.66 – 0.89	0.77
**R^2**	**F-statistic**			
0.0035	0.087			

Table S10. Table showing the predictor variable and the estimate with standard error, confidence intervals and P values for linear regression models to study *AmIlp1* expression levels in nurses and foragers in *A. florea* and *A. cerana*. *AmIlp1* levels were significantly higher in foragers than nurses.

(a) *A. florea*

**Predictor**	**Estimate**	**Std. Error**	**CI**	**P value**
(Intercept)	-0.56	0.20	-0.98 – -0.15	0.0096**
Social.Role.F	1.13	0.29	0.54 – 1.72	0.0005***
**R^2**	**F-statistic**			
0.356	15.47			

(b) *A. cerana*

**Predictor**	**Estimate**	**Std. Error**	**CI**	**P value**

Journal of Experimental Biology • Supplementary information

(Intercept)	-0.58	0.22	-1.01 – -0.15	0.014*
Social.Role.F	1.09	0.30	0.5 – 1.68	0.0013**
**R^2**	**F-statistic**			
0.33	12.9			

Table S11. Table showing the predictor variable and the estimate with standard error, confidence intervals, R^2, F-statistic and P values for linear regression models to study 4 TFs, *usp, Egr -1, BR-C* and *nautilus* expression levels in nurses and foragers in *A. florea*. All TFs except for nautilus were higher in foragers than nurses. *Nautilus* wasn't different between nurses and foragers.

(i) *Usp*

**Predictor**	**Estimate**	**Std. Error**	**CI**	**P value**
(Intercept)	-0.54	0.21	-0.97 – -0.11	0.015*
Social.Role.F	1.08	0.29	0.48 – 1.68	0.001**
**R^2**	**F-statistic**			
0.32	13.4			

(ii) *Egr - 1*

**Predictor**	**Estimate**	**Std. Error**	**CI**	**P value**
(Intercept)	-0.74	0.16	-1.07 – -0.42	<0.0001****
Social.Role.F	1.49	0.22	1.06 – 1.92	<0.0001****
**R^2**	**F-statistic**			
0.61	44.7			

(iii) *BR-C*

**Predictor**	**Estimate**	**Std. Error**	**CI**	**P value**
(Intercept)	-0.46	0.22	-0.91 – -0.003	0.048*
Social.Role.F	0.92	0.31	0.27 – 1.56	0.007**
**R^2**	**F-statistic**			
0.23	8.5			

Journal of Experimental Biology • Supplementary information

(iv) *Nautilus*

**Predictor**	**Estimate**	**Std. Error**	**CI**	**P value**
(Intercept)	-0.32	0.24	-0.81 – 0.17	0.19
Social.Role.F	0.64	0.34	-0.06 – 1.33	0.07
**R^2**	**F-statistic**			
0.11	3.5			

Table S12. Table showing the predictor variable and the estimate with standard error, confidence intervals, R^2, F-statistic and P values for linear regression models to study 4 TFs, *usp, Egr -1, BR-C* and *nautilus* expression levels in nurses and foragers in *A. cerana*. All TFs except for nautilus were higher in foragers than nurses. *Nautilus* wasn't different between nurses and foragers.

**Predictor**	**Estimate**	**Std. Error**	**CI**	**P value**
(Intercept)	-0.55	0.23	-1.02 – -0.08	0.023*
Social.Role.F	1.03	0.31	0.39 – 1.67	0.003**
**R^2**	**F-statistic**			
0.29	10.8			

(ii) *Egr - 1*

**Predictor**	**Estimate**	**Std. Error**	**CI**	**P value**
(Intercept)	-0.77	0.18	-1.13 – -0.41	0.0001***
Social.Role.F	1.44	0.24	0.95 – 1.93	<0.0001****
**R^2**	**F-statistic**			
0.58	35.8			

(iii) *BR-C*

**Predictor**	**Estimate**	**Std. Error**	**CI**	**P value**
(Intercept)	-0.43	0.25	-0.94 – 0.08	0.09
Social.Role.F	0.80	0.34	0.11 – 1.5	0.02*
**R^2**	**F-statistic**			
0.18	5.7			

Journal of Experimental Biology • Supplementary information

(iv) *Nautilus*

**Predictor**	**Estimate**	**Std. Error**	**CI**	**P value**
(Intercept)	-0.34	0.26	-0.87 – 0.19	0.2
Social.Role.F	0.63	0.35	-0.09 – 1.35	0.08
**R^2**	**F-statistic**			
0.11	3.2			

Journal of Experimental Biology • Supplementary information
